# Guillain-Barré syndrome as clinical presentation of a recently acquired hepatitis C

**DOI:** 10.1007/s13365-023-01167-7

**Published:** 2023-08-30

**Authors:** Filomena Boccia, Letizia Lucia Florio, Emanuele Durante-Mangoni, Rosa Zampino

**Affiliations:** 1 Department of Advanced Medical and Surgical Sciences, University of Campania “L. Vanvitelli”, Naples, Italy; 2 Unit of Infectious & Transplant Medicine, AORN Ospedali dei Colli, Monaldi Hospital, Naples, Italy; 3 Department of Precision Medicine, University of Campania “L. Vanvitelli”, Naples, Italy

**Keywords:** Post-viral syndrome, Paralysis, Serology, Viremia, Diagnosis

## Abstract

About 40% of the Guillain-Barré syndrome (GBS) cases are associated with prodromal infections; occasionally, it has been associated to chronic hepatitis C or its reactivation. A 38-year-old man came to our attention after transaminase elevation occurred during recovery from GBS. All the possible causes of acute hepatitis were excluded except for the positivity of HCVRNA, and a diagnosis of new onset hepatitis C was made. Recalling patient history, we observed that (i) anti-HCV antibodies were negative and liver enzymes were normal 7 weeks before GBS onset; (ii) in the early stages of ICU admission, liver enzymes started to rise, but the elevation remained mild under steroid treatment; (iii) serum aminotransferase peak occurred 11 weeks after GBS onset; and (iv) HCV RNA was already significantly high when anti-HCV antibodies became positive, consistent with an acute hepatitis. Furthermore, anti-HCV seroconversion was likely delayed or blurred by steroids and immunoglobulin infusions. The interval of time between GBS onset and transaminase elevation compared with the patient clinical history allows us to establish a cause-effect relationship between the two diseases. All patients with GBS should be tested for hepatitis C, or its reactivation if already present, and followed up for an early diagnosis and treatment.

## Introduction

The Guillain-Barré syndrome (GBS) is an immune-mediated demyelinating polyradiculoneuropathy with various clinical presentations and increasing incidence (Shahrizaila et al. 2021). In most cases, a causal link with a respiratory or intestinal infection in the previous 6 weeks could be established. Several viruses and bacteria have been linked to GBS (Shahrizaila et al. 2021).

Hepatitis C virus (HCV) infection has been associated with extrahepatic manifestations, often mediated by aberrant immune responses or cryoglobulins (Cacoub et al. 2021). Few cases of GBS associated with chronic hepatitis C (CHC) or HCV reactivation have been reported (Chlilek et al. 2019; Lacaille et al. 1998).

We describe a case of a recently acquired HCV infection, whose acute clinical onset was heralded by GBS.

## Case report

A 38-year-old man was admitted to an outside hospital intensive care unit because of fever associated to gastrointestinal symptoms and progressive centripetal muscle weakness, rapidly evolving into flaccid tetraplegia, absent reflexes response, and paresthesias combined with dysphagia and dysphonia. Electroneuromyography showed a pattern diagnostic for demyelinating motor polyneuropathy with proximal and distal conduction blocks, consistent with GBS. Cerebral spinal fluid exam and tests for anti-gangliosides antibodies were not performed. Corticosteroids and intravenous human immunoglobulins at the dose of 2 g/kg were administered, followed by physiotherapy for the next 2 months.

During rehabilitation, a mild aspartate aminotransferase (AST) and alanine aminotransferase (ALT) elevation was observed; AST and ALT levels progressively and constantly increased (13.8 × upper normal limit (UNL) and 16.2 × UNL, respectively) while HBsAg and anti-HCV antibodies were negative.

Within the next 6 days, liver enzymes almost doubled (AST 23.8 × UNL, ALT 34.3 × UNL, γ-glutamyl transferase 3.6 × UNL), ferritin peaked at 3024 mg/dl, while C-reactive protein, procalcitonin, white blood cells, red blood cells, platelets, and kidney function tests remained normal.

The patient was referred to our unit for further investigation. He complained of severe weakness, difficulty to stand up, and a slowed gait. He denied assumption of foods, drugs, or teas known to be hepatotoxic. Clinical examination showed mild hepatomegaly, a residual lower limb weakness, and finger tips dysesthesia, while there was a complete recovery of dysphagia, dysphonia, and reflexes response. Reported comorbidities included a treated Hashimoto’s thyroiditis and Gilbert’s disease.

Because of the possible accidental job-related exposure to materials contaminated with biologic samples, we analyzed all available tests the patient did in the previous years: HBsAg and anti-HCV antibodies resulted absent until 7 weeks before GBS onset.

A detailed work-up for diagnosis of acute and chronic hepatitis was performed: tests for hepatis viruses type A, B, C, D, E; HAV, HCV, HBV, HEV, cytomegalovirus, Epstein-Barr virus, herpes simplex virus 1 and 2; autoimmune liver markers (anti-mitochondrial antibodies, antinucleous antibodies, liver-kidney microsomal antibodies type 1, anti-smooth muscle antibodies, anti-neutrophil cytoplasma antibodies, cryoglobulins), α-ceruloplasmin, α-1-antitripsin, iron status assessment. Among these, only HAV IgG and anti-HCV antibodies were positive.

HCV-RNA level was 1.06 × 10^7^ IU/ml, HCV genotype 3a, while ALT peaked at 46.8 × UNL and AST at 28.8 × UNL, remaining persistently elevated. Bilirubin, mainly indirect, was slightly elevated and other liver function tests were normal.

Liver ultrasound was normal and liver elastography reported a stiffness of 6.9 kPa (fibrosis stage 1).

**Fig. 1:**
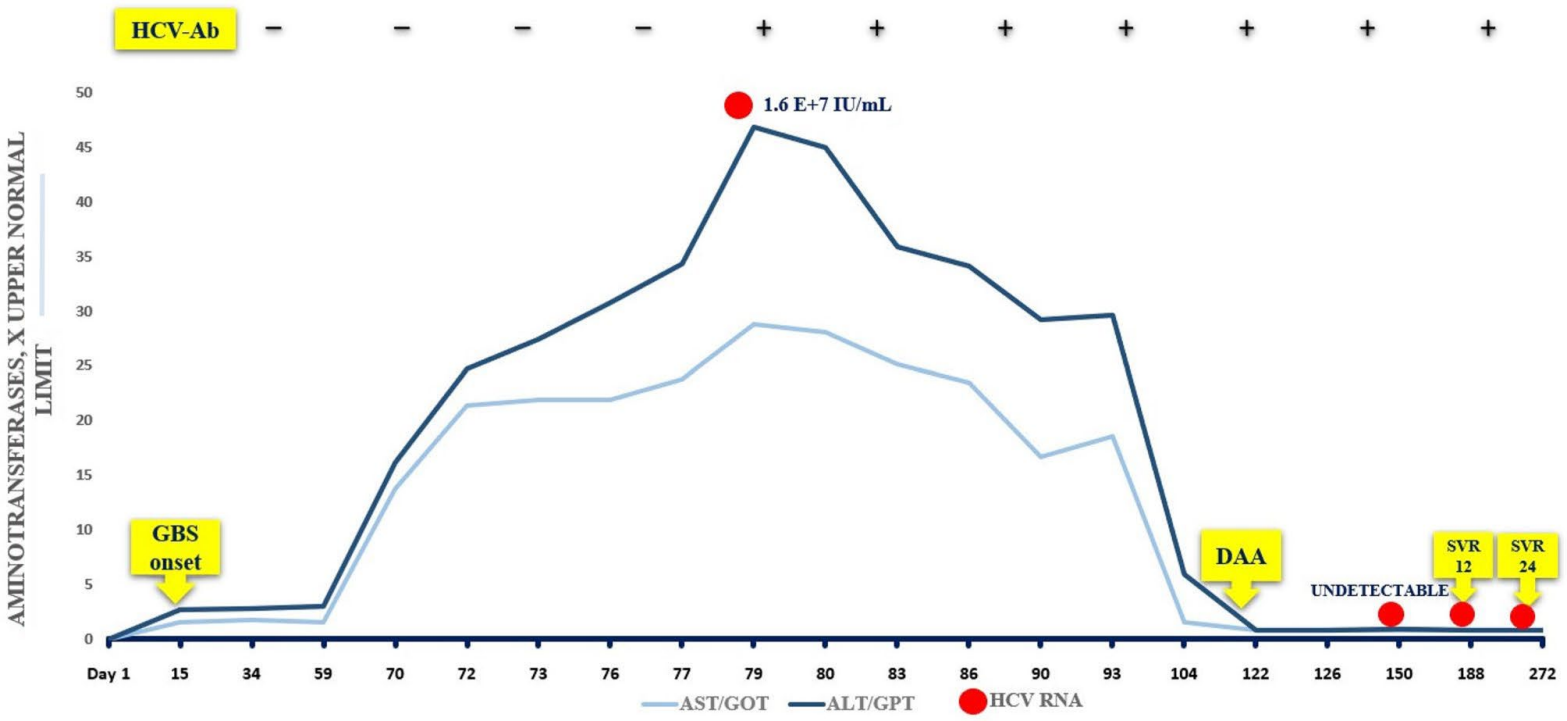
Clinical course of HCV infection over time

Antiviral therapy with sofosbuvir/velpatasvir (400/100 mg/day for 12 weeks) was started, with rapid drop of ALT and AST; after 1 month of treatment, AST and ALT were normal and serum HCV RNA became undetectable. HCV RNA remained undetectable during the 24 weeks following the end of treatment, showing a sustained viral response (SVR 24) (Fig. 1).

Patient gave his written informed consent to the processing and publication of his clinical data.

## Discussion

Peripheral nervous system involvement in chronic HCV infection is a well-known complication, mainly due to an immune-mediated response (Chlilek et al. 2019; Lacaille et al. 1998; Balwani et al. 2018). A molecular mimicry could trigger the GBS autoimmune demyelination, as described for other microorganisms (Rodriguez et al. 2019; Faccioli et al. 2021).

Clinical cases of acute viral hepatitis associated to GBS have been described for HAV and HEV (Oh et al. 2017; Tse et al. 2012), while the association with CHC has been reported in cases of HCV reactivation (Malhotra et al. 2022; Macleod et al. 1987).

To our knowledge, only 4 cases have been associated to acute/subacute HCV (Macleod et al. 1987, DeKlippel et al. 1993; Belbézier et al. 2019), but some of them have been described before the discovery of HCV (non-A non-B hepatitis) or immediately thereafter. The most recent case was described by Belbézier in 2019 in a 73-year-old man on treatment for HIV infection (Belbézier et al. 2019).

Although the authors of the previous cases interpreted them as an early phase of an acute hepatitis C, this remains uncertain: a flare of aminotransferases was not described and previous HCV infection was not clearly ruled out.

We present a clinical case that temporally relates documented acute hepatitis C to the onset of GBS.

Recalling patient history, we could observe that (i) anti-HCV antibodies were negative and liver enzymes normal 7 weeks before GBS onset; (ii) in the early stages of ICU admission, liver enzymes started to rise, but the elevation remained mild under steroid treatment; (iii) ALT peak occurred 11 weeks after GBS onset; (iv) HCV RNA was already high when anti-HCV antibodies became positive, as in acute hepatitis; and (v) anti-HCV seroconversion was likely delayed or blurred by steroids and immunoglobulin infusions.

It is plausible to assume that progression of neurological symptoms and hepatitis matched ab initio and that HCV infection has been the trigger for GBS; steroids administered for GBS treatment probably facilitated HCVRNA level increase. Anti-HCV antibodies had been negative for 11 weeks, so an earlier search for serum HCVRNA could possibly allow a more timely diagnosis of HCV infection.

A prompt evaluation of HCV infection in patients with GBS appears important for patient clinical care and reducing risk of HCV transmission. An accurate evaluation of clinical history can reduce the delay in HCV infection diagnosis and HCVRNA should be performed in patients with GBS and elevated ALTs, particularly when drug-induced immunosuppression occurs and no other specific causes of GBS become apparent.

## Conclusion

We provide evidence to suggest that GBS can be a presenting syndrome of acute hepatitis C. Patients diagnosed with GBS showing elevated aminotransferases should be screened for HCV infection.
